# Superinfection Exclusion in Mosquitoes and Its Potential as an Arbovirus Control Strategy

**DOI:** 10.3390/v12111259

**Published:** 2020-11-05

**Authors:** Mathilde Laureti, Prasad N. Paradkar, John K. Fazakerley, Julio Rodriguez-Andres

**Affiliations:** 1 Peter Doherty Institute for Infection and Immunity and Faculty of Veterinary and Agricultural Sciences, University of Melbourne, VIC 3000 Melbourne, Australia; john.fazakerley@unimelb.edu.au; 2 CSIRO Health & Biosecurity, Australian Centre for Diseases Preparedness, VIC 3220 Geelong, Australia; Prasad.Paradkar@csiro.au

**Keywords:** superinfection exclusion, arbovirus, vector control

## Abstract

The continuing emergence of arbovirus disease outbreaks around the world, despite the use of vector control strategies, warrants the development of new strategies to reduce arbovirus transmission. Superinfection exclusion, a phenomenon whereby a primary virus infection prevents the replication of a second closely related virus, has potential to control arbovirus disease emergence and outbreaks. This phenomenon has been observed for many years in plants, insects and mammalian cells. In this review, we discuss the significance of identifying novel vector control strategies, summarize studies exploring arbovirus superinfection exclusion and consider the potential for this phenomenon to be the basis for novel arbovirus control strategies.

## 1. Introduction

Over the past century, there has been continual emergence and re-emergence of arbovirus diseases, with more than 80% of the world’s population at risk of infection [1]. Arboviruses (Arthropod-Borne viruses) include mosquito-borne viruses, such as yellow fever (YFV), Zika (ZIKV), West Nile (WNV), Japanese encephalitis (JEV), dengue (DENV) and chikungunya (CHIKV) viruses, and tick-borne viruses such as tick-borne encephalitis virus (TBEV) [2]. Globally, there are over 100 arbovirus diseases of human and veterinary health concern [3]. The epizootic transmission and pandemic potential of arboviruses are affected by several factors including climate change, population growth, urbanization and intercontinental travel and trade [2,4]. For example, both global warming and human travel and trade are expanding the range of viral vectors like the *Aedes spp.* mosquitoes [5]. Several strategies are being used and developed to counter the spread of these viruses by mosquitoes. These include the use of pesticides, infection with the bacterium *Wolbachia* and the use of genetically modified mosquitoes. In this review, we will discuss superinfection exclusion as an alternative strategy. This strategy is based on the premises that if a mosquito is infected with a non-pathogenic virus, a secondary infection with a related pathogenic virus is less likely, reducing or preventing the transmission of the second infection.

## 2. Arboviruses

Arboviruses are found across several virus groups including the single-stranded, positive-sense RNA viruses of the Flaviviridae and Togaviridae, the segmented negative-sense RNA Bunyaviridae and the segmented double-stranded (ds)RNA viruses of the Reoviridae.

The Flaviviridae family contains over 70 arboviruses including some of the most medically important ZIKV, DENV, JEV, WNV and YFV [6]. They include both mosquito-borne and tick-borne viruses [7,8]. More than 40 flaviviruses have been associated with human disease, and the number is expected to grow [2]. Some flaviviruses have more than one serotype, increasing the number of known human pathogens. For example, there are four major antigenically distinct DENV serotypes, DENV-1–4, with a fifth of them under investigation [9,10]. In 2012, at least one of the four DENV serotypes was reported in over 120 countries, placing approximately 50% of the world’s population at risk [11]. After causing global outbreaks, ZIKV is currently circulating in more than 87 countries around the world according to WHO’s latest epidemiological update. However, ZIKV infection remains asymptomatic in 80% of cases, with rare severe clinical outcomes such as microcephaly and Guillain–Barré syndrome [12]. JEV, one of the major causes of viral meningoencephalitis in the world, has been reported in at least 24 countries, endangering more than 3 million people, and YFV is still detected in 40 countries despite the availability of an effective vaccine since the 1930s [13,14].

The *Alphavirus* genus within the Togaviridae family is generally divided into groups based on clinical symptoms. The arthritogenic viruses include CHIKV, Sindbis (SINV) and Ross River (RRV) viruses. The encephalitic viruses include Eastern equine encephalitis (EEEV), Venezuelan equine encephalitis (VEEV), and Western equine encephalitis (WEEV) viruses. Phylogenetically, the *Alphavirus* genus includes an aquatic virus clade encompassing salmon pancreas disease virus and sleeping disease virus, a Sindbis clade (SINV), an equine encephalitis clade (VEEV, EEV and WEEV) and a Semliki Forest clade (SFV, RRV, CHIKV) [15]. CHIKV infection is normally not life-threatening but is responsible for high morbidity with painful and long-term symptoms, such as crippling arthralgia that may require hospitalization, but it can cause fatal encephalopathy, encephalitis and multiple-organ failure [16]. The virus is endemic in Africa where it is vectored by *Aedes aegypti*. A single amino acid change in the viral envelope E1 protein allowed its adaptation to *Aedes albopictus*, leading to the global expansion of CHIKV, with far-reaching consequences [17]. From 2005 to 2006, more than 1.5 million cases were reported in the Indian Ocean, with subsequent spread in 2007 to Italy and France [4,18]. During the epidemic on La Reunion Island, one-third of the island population was infected by CHIKV and hospitalized within six months, at a medical cost of €44 million [19]. In 2013–2016, there were outbreaks in the Caribbean and Brazil [20]. In 2016, CHIKV was responsible for more than 1 million infections in the Americas [21]. SINV is the alphavirus with the widest global distribution and can be found on most continents [22]. SINV strains isolated in Europe have been linked to Pogosta disease in Finland, Ockelbo disease in Sweden and Karelian fever in Russia, three clinically similar diseases characterized by polyarthritis, myalgia, headache, low fever and rash [23,24,25]. RRV is another arthritogenic alphavirus transmitted by both *Aedes* and *Culex* mosquitoes. It is endemic to Australia, with approximately 5000 new cases per annum [26]. Outbreaks have also been recorded in Fiji, New Caledonia, Samoa and the Cook Islands with tens of thousands of infections [27,28,29].

Since many of these flaviviruses and alphaviruses are transmitted by the same mosquito vectors, overlapping outbreaks of these viruses have been recorded. For example, during 2016–2017, overlapping outbreaks of CHIK, DENV and ZIKV were reported in 36 countries, associated with *Ae. aegypti* transmission [30]. Newly emerging arboviruses including Mayaro virus [31], African swine fever virus [32] and new strains of bluetongue and Usutu viruses [33] represent threats to human and animal health [34]. With the exception of YFV, DENV and JEV, there are no approved vaccines available for human clinical use for arboviruses, and vector control remains an important strategy to prevent these diseases.

### 2.1. Insect-Specific Viruses

Insect-specific viruses (ISVs), i.e., viruses that replicate solely in insects, have been found so far in both the *Flaviviridae* and the *Togaviridae* families. These viruses cannot infect vertebrates and therefore are not zoonotic. Over the last decade, with the increasing use of high-throughput sequencing driving virus discovery, increasing numbers of ISVs have been discovered in mosquitoes [35].

The first discovered insect-specific flavivirus (ISFV), i.e., cell-fusing agent virus, was found in *Ae. aegypti* mosquito cell lines and named after the syncytia it forms in these cells [36]. In 2003, another ISFV, the Kamiti River virus, was isolated from *Aedes mcintoshi* mosquitoes in central Kenya [37]. In 2007, a *Culex*-specific flavivirus was isolated from mosquitoes in Japan [38]. More sequence-related ISFVs have since been discovered around the world: *Aedes*-specific flaviviruses in Japan, Italy and the USA, Nakiwogo virus in Uganda, Calbertado virus in Colorado (USA), Quang Binh virus in Vietnam, Hanko virus in Finland, Palm Creek virus in Australia and many more [39,40,41,42,43,44,45,46]. ISFVs are phylogenetically classified in two groups [47]. One of those groups is clearly distinct from any other known flavivirus and includes cell-fusing agent virus, Kamiti River virus and Culex flavivirus. The second group is related to vertebrate flaviviruses and includes Chaoyang virus, Nounané virus and Lammi viruses. Molecular phylogeny studies estimate that there may be in the order of 2000 ISFVs to be discovered [48].

Unlike ISFVs, only a few insect-specific alphaviruses (ISAV) have been discovered so far: Eilat virus (EILV), Taï Forest alphavirus (TAFV), Mwinilunga virus (MWAV), Agua Salud virus (ASALV) and Yada Yada virus (YYV) [49,50,51,52,53]. TAFV was discovered in the Taï national park in the Ivory Coast in *Culex decens* mosquitoes [49]. MWAV was isolated from a pool of *Culex quinquefasciatus* mosquitoes from Zambia [50]. ASALV and YYV are the most recently discovered ISAVs. ASALV was isolated from a pool of *Culex* mosquitoes in Central Panama, and YYV was isolated from *Anopheles annulipes* mosquitoes in Australia [51,53]. EILV, the first ISAV described, was isolated from a pool of *Anopheles coustani* mosquitoes collected near Jerusalem in 1984 [54]. EILV does not infect mammalian cells or mouse brain, a tissue which has been historically used to amplify or isolate many viruses [52]. *Aedes*, *Anopheles* and *Culex* mosquitoes are susceptible to EILV infection. However, *Ae. albopictus* seems to be refractory to oral infection, while *Ae. aegypti* is susceptible to both oral and intrathoracic infection [55].

Insect-specific viruses seem to have potential as an arbovirus control method in several ways, from making mosquitoes refractory to related arboviruses to reducing mosquito fitness and fertility [56,57]. However, more research about their transmission and specificity is necessary to their enable large-scale implementation [58].

### 2.2. Current Control Stategies

The main vector control strategies for arboviruses include larvicides, insecticides, suppression of breeding sites, bed nets and insect repellents. Each of these has limitations. Mosquitoes such as *Ae. aegypti* can breed in a small volume of water, and suppression of every breeding site is unrealistic. *Ae. aegypti* and *Ae. albopictus* are day biters, so bed nets have little efficacy. Mosquito resistance to insecticides is increasing worldwide, and with high costs and environmental damage, its usage is unsustainable, in addition to the lack of evidence of the effect of pesticides on areas with outbreaks [59]. New control strategies are needed to control mosquito-borne diseases.

### 2.3. Novel Control Strategies

Several new vector control strategies are under investigation. The environmentally friendly, naturally occurring symbiotic *Wolbachia* bacteria found in many insects have emerged as a new effective vector control mechanism. Mating of *Wolbachia*-infected male mosquitoes with uninfected wild female mosquitoes leads to sterile eggs and population suppression, whereas mating between *Wolbachia*-infected female mosquitoes and infected or uninfected male mosquitoes gives rise to fertile eggs and offspring [60,61]. Through this mechanism, *Wolbachia* infection is driven into a mosquito population. So far, 18 supergroups of Wolbachia have been discovered in the wild [62]. There are now multiple studies showing that *Wolbachia* infection of female mosquitoes prevents the transmission of DENV and CHIKV [61,63,64,65,66,67,68,69]. Field studies are underway to determine whether driving *Wolbachia* into mosquito populations in areas endemic for DENV can reduce the incidence of dengue fever; results to date are promising [66,67,68,70,71,72,73]. Further studies are required to fully understand the mechanism by which *Wolbachia* prevents arbovirus transmission and to understand the ecological effects of introducing this bacterium into wild *Ae. aegypti* mosquito populations, which are not naturally infected [59]. There is also concern that *Wolbachia* infection of mosquitoes might drive the evolution of viruses with increased infectivity or virulence [74].

Other novel systems to control arbovirus transmission involve the genetic modification (GM) of mosquitoes [75]. The sterile insect technique has previously been used for decades to prevent the transfer of new-world screwworm blowflies from south to north America [76]. Large numbers of insects are bred, radiation-sterilized and released. Wild females mated with sterile males lay no eggs, resulting in a crash in the blowfly population. Studies are underway to replace irradiation with genetically modified conditionally sterile males [77]. Other GM approaches include creating mosquitoes carrying a genetic payload system which can be transmitted to the female progeny, which renders these female mosquitoes resistant to a specific arbovirus infection [78]. Manipulating mosquito immunity to induce death upon infection can be argued to be more effective than resistance to infection, since the former is less likely to drive virus evolution [79]. These technologies rely on recent advances in mosquito genetics as well as on CRISPR–Cas9 technology [80]. The ability to drive genetic change into a population while ensuring it does no lasting ecological damage is a challenge. GM mosquitoes are currently being developed and studied in laboratories around the world. GM mosquito technology is relatively new, but as with *Wolbachia*, field trials are currently underway. In 2016, billions of GE OX513A *Ae. aegypti* mosquitoes were released in areas of Brazil with a high incidence of DENV and ZIKV cases. *Ae. aegypti* OX513A is a genetically engineered strain carrying a self-limiting gene which renders mating between OX513A males and wild-type females unproductive [81]. Initial studies from Grand Cayman, Brazil and Panama have reported significant reductions in vector abundance. *Ae. aegypti* OX5034 are male-selective, and mating between OX5034 and wild-type females results in the survival of only the male progeny [82]. The release of OX5034 has recently been approved for use in Florida to reduce the incidence of dengue [81,82,83]. The use of both *Wolbachia* and GM mosquitoes requires extensive regulation and public engagement, but both technologies have good potential to control arbovirus outbreaks [84].

## 3. Superinfection Exclusion

Superinfection exclusion is observed when a first infection prevents the replication of a second infection by a genetically identical or a genetically closely related virus; this phenomenon is also termed homologous and heterologous interference, respectively. Superinfection exclusion could be another arbovirus control strategy.

Interference between plant viruses has been recognized and studied for almost a century [85,86,87]. Cross-protective interference has been developed in agriculture and horticulture to protect plants from pathogenic viruses by deliberate prior infection with an attenuated strain of the same virus [88]. Recent decades have witnessed enormous progress in understanding plant immunity, which principally works through the recognition of pathogen-associated molecular patterns (PAMPs) by pattern recognition receptors (PRR) on the cell surface and within the cell [89,90]. dsRNA is a PAMP associated with RNA virus infections. Cellular RNA is single-stranded, but viruses with an RNA genome must replicate their RNA and, in doing so, they form dsRNA. The RNA interference (RNAi) plant immunity system detects and destroys the viral RNA. Virus dsRNA is detected by the PRR dicer-like protein (DCL). This cleaves the virus dsRNA into small RNAs, one strand of which is then incorporated into an RNA-induced silencing complex (RISC). This in turn silences, that is, degrades, sequence-complementary RNA [91]. While plant immunity, and RNAi in particular, may explain the described interference between many plant viruses, which has in some cases been termed superinfection exclusion, there is evidence that other mechanisms, related not to host systems but to properties of the virus, may also be at work [87].

In mammals, the innate and adaptative immune systems provide protection against sequential infections between distantly and closely related viruses. In mammalian cell culture, as in plants, the term superinfection exclusion has been used over many decades to denote the phenomenon by which one virus infection precludes the replication of a second infection by a related virus [92,93,94]. The powerful antiviral interferon system, a component of innate immunity in mammals, provides highly effective cross-protection between even unrelated viruses, at least for a short period after primary infection [95]. While some cases termed superinfection exclusion in mammalian cells can probably be attributed to interferons or other aspects of innate immunity, other mechanisms of exclusion also appear to be operating [96,97,98].

In arthropods, virus co-infection and superinfection exclusion have been studied in mosquitoes and mosquito cells for many years [99] (Table 1). Both homologous and heterologous interference are observed in *Aedes* mosquito cells persistently infected with SINV [100]. That is, once infected with SINV, the cells cannot be superinfected with other strains of SINV or other alphaviruses [100,101,102]. However, these same cells can be superinfected with unrelated bunyaviruses or the flavivirus YFV [102]. Heterologous interference is also observed in insect cells infected with flaviviruses, for example with YFV- in DENV-2-infected *Ae. albopictus* cells [99]. In mosquitoes, several studies have demonstrated that sequential infection by viruses of different families results in coinfection, not in exclusion [103,104,105].

## 4. Mechanisms of Superinfection Exclusion

From the point of view of virus evolution, superinfection exclusion could be considered a selective advantage mediated by a virus-encoded mechanism that functions to reduce competition for limited cellular resources by preventing entry into the cell, or replication within the cell, of additional viruses [111]. This phenomenon is mostly observed between closely related viruses, suggesting that competition for resources, interference between virus-derived molecules or activation of specific cellular defenses may play important roles in this event [112]. Interestingly, however, there are cases when interference might occur between different viral families. Menghai rhabdovirus (MERV) and Shinobi tetravirus (SHTV) infection of *Ae. Albopictus*-derived C6/36 cells suppressed superinfected ZIKV growth [113]. In addition, the opposite can also occur with unrelated viruses, for example the *Ae. aegypti* totivirus (AaTV) from the Totiviridae family has shown to boost superinfecting DENV-1 production in C6/36 cells [114]. The mechanisms of this interaction remain unknown [111].

In mammalian cells, superinfection exclusion has been reported to occur at virus entry or upon virus replication [115,116,117,118]. Superinfection exclusion can be established rapidly after primary infection. Studies on SINV and SFV heterologous interference show that cellular DNA-dependent transcription is not required for superinfection exclusion to function, suggesting it is not interferon-mediated [92]. SFV infection of BHK-21 cells showed that 15 min of primary infection was sufficient to establish exclusion by a superinfecting virus and that the second virus was unable to bind to the pre-infected cells [119]. In Madin–Darby bovine kidney (MDBK) epithelial cells infected with the flavivirus bovine viral diarrhea virus (BVDV), superinfection exclusion was functional in less than an hour, consistent with a block at virus entry [117]. Competition for binding to cellular receptors and the decreased number of cell surface receptors available after primary infection could be the mechanism mediating interference for the second infection [119]. Given the specificity of receptor binding, this would be consistent with exclusion only between related viruses.

In the case of the two viruses considered above, bypassing virus cell surface receptors by transfecting virus RNA into pre-infected cells did not prevent exclusion by SINV in BHK-21 cells or by BVDV in MDBK cells [117,118]. In addition to interference with second virus entry, there are several potential points in virus replication within the cell at which the products of a first infecting virus could interfere with the replication of a related second incoming virus. To consider this requires an understanding of virus replication; alphaviruses provide an example. Upon entry into the cytosol, alphavirus nucleocapsids bind to ribosomes. This triggers nucleocapsid disassembly and release of the virus genomic RNA [120]. This provides potential for a first virus to prevent infection of a second through saturation by the first virus of the capsid protein binding sites on ribosomes, preventing nucleocapsid binding and disassembly of a second virus infection [121]. However, in BHK-21 cells infected with SFV, superinfection exclusion still occurred when the primary infection was a recombinant virus that produced no capsid protein [119]. Upon release of the virus genome from the nucleocapsid, alphavirus replication starts with the translation of the non-structural polyprotein (ns) P1234 encoded at the 5′ end of the virus genomic RNA. A second open reading frame (ORF), translated from a subgenomic RNA, encodes for the structural polyprotein [122]. The alphavirus structural proteins are involved in viral assembly, release, transmission, attachment and entry. The components of the non-structural polyprotein mediate replication and transcription of the virus RNA. P1234 is processed into four mature non-structural proteins (nsP) 1–4. The functional proteins are separated by the activity of a protease located in nsP2. Processing leads to sequential release of nsP4 and nsP1 and then separation of nsP2 and nsP3. The extent of processing determines the substrate specificity of the replicase complex which switches from synthesis of negative-stranded genomic complementary RNA to synthesis of subgenomic and genomic RNA upon cleavage at the nsP2–nsP3 junction [123,124]. SFV nsP1 does not mediate superinfection exclusion, since cells expressing it do not block subsequent SFV entry or replication [125]. The nsP2 protease has been suggested to mediate superinfection exclusion on the basis that the replicase polyprotein synthesized from a new alphavirus genome entering a pre-infected cell could be rapidly processed by existing nsP2 protease. The fully processed replicase produces genomic RNA from a genome complementary negative-sense RNA substrate. The rapid processing of the new replicase polyprotein could result in no negative-stranded template and thus no genome replication of the second virus [102]. Attempts to investigate this by expressing nsP2 have been unsuccessful, as this virus protein, expressed in the absence of the other replicase proteins, is toxic to mammalian cells [126]. For the flavivirus WNV, a mutation in the non-structural protein NS4A of WNV-infected BHK-21 cells allowed superinfecting wild-type WNV to overcome exclusion [127].

In arthropods, as in plants, dsRNA formed by RNA virus replication in the cytoplasm activates the cellular RNAi defense system, which generates specific immunity that degrades RNAs containing identical sequences. The system consists of a number of related pathways; 21 nucleotide sequence identities form the basis of the siRNA pathway, while larger nucleotide identities of up to 30 nucleotides form the basis of the piRNA pathway. For RNAi to be cross-protective between viruses, therefore, regions of RNA sequence identity from 21 to 30 nucleotides are required. Alignment of the 11 kb alphavirus genomes of CHIKV and SINV demonstrates no 21–30 nucleotides sequence identities. *Ae. albopictus* C6/36 and C7/10 cells lack a functional dicer-2 protein and siRNA pathway but have a functional piRNA pathway [128,129]. Upon virus infection, these cells demonstrate no 21nt siRNA response and a delayed piRNA response but remain efficient at superinfection exclusion, suggesting that the siRNA pathway is not required but not ruling out its contribution [102,130].

The alphavirus genome encodes five structural proteins: the capsid, the envelope proteins E1, E2 and E3, as well as the 6K envelope protein [131]. The structural polyprotein is cleaved into these functional proteins by viral and cellular proteases [132]. The capsid is cleaved first, leaving the polyprotein precursor E3–E2–6K–E1 which is processed by the cellular signalase protease into 6K, E3E2 (PE2) and E1 [133]. E2 is responsible for receptor-mediated endocytosis, while E1 mediates fusion of the viral membrane with that of the endolysosome [134,135]. The 6K protein has a transmembrane domain involved in ion-channel activity and another transmembrane domain which interacts with E1 [131]. In SFV-infected BHK-21 cells, virus inability to produce capsid protein did not prevent superinfection exclusion [119]. For WNV, the absence of structural proteins (C, M and Env) did not significantly affect heterologous interference with the related flaviviruses YFV or DENV-2; however, St. Louis encephalitis virus (SLEV) titers were severely hindered by the expression of the WNV nonstructural replicon alone [136]. In contrast, in MDBK cells harboring recombinant BVDV with deletions of E2, E1, or C proteins, deletion of E2, but not of E1 or C, abolished subsequent superinfection exclusion [117]. The BVDV E2 protein is essential for the generation of new infectious virions, but how the E2 protein mediates superinfection exclusion remains unclear [117,137].

## 5. Superinfection Exclusion as an Arbovirus Control Strategy

The persistent emergence and re-emergence of mosquito-transmitted viral diseases, such as chikungunya, dengue and Zika, warrants new arbovirus control strategies. As with yellow fever, vaccination is likely to be powerful and important. Dengue vaccines have been recently approved, and chikungunya and new Japanese encephalitis vaccines are under development. In the control of mosquito transmission, *Wolbachia* infection and genetically modified mosquitoes show promise. The relatively recent discovery of insect-only alphaviruses and flaviviruses suggests that, as for *Wolbachia* infection, an insect-only arbovirus might effectively preclude infection with, or transmission of, a related zoonotic arbovirus.

Many zoonotic arboviruses share mosquito vector species, and spatially and temporally overlapping outbreaks of arbovirus disease are well documented [30]. For example, in recent decades, many of the outbreaks of chikungunya and Zika have occurred in areas with a high ongoing incidence of dengue. Reports that mosquitoes can be naturally infected with more than one arbovirus include *Ae. aegypti* co-infection with ZIKV and CHIKV and with ZIKV, CHIKV and DENV, and *Ae. albopictus* co-infection with DENV and CHIKV [103,104,138]. Co-infections have also been observed in artificially fed *Ae. aegypti* with DENV and CHIKV as well as triple infection with CHIKV, DENV and ZIKV [138]. In contrast to these co-infections with genetically unrelated or distantly related viruses, *Ae. aegypti* infected with one DENV serotype have decreased susceptibility to infection with other serotypes [110]. In another study of *Ae. Aegypti* experimentally infected with DENV and ZIKV, the mosquitoes became co-infected, but only ZIKV was present in saliva [139]. In a rare insight into what may be happening in ticks, in a screening for virus infection of *Ixodes scapularis* ticks, no co-occurrence was observed between two different strains of Blacklegged tick phlebovirus, suggesting exclusion between these viruses [140].

There is evidence that insect-only arboviruses can prevent infection or transmission of a related zoonotic arbovirus. Studies on the mosquito-specific alphavirus EILV demonstrated both homologous and heterologous interference in *Ae. albopictus* cells [52]. EILV infection blocks superinfection of a different strain of EILV or different alphaviruses including SINV, VEEV, EEV, WEEV and CHIKV [108]. This same study also investigated heterologous interference in *Ae. Aegypti*, a mosquito susceptible to both EILV and CHIKV infection. Mosquitoes were intra-thoracically injected with EILV and blood-fed 7 days later with CHIKV. Absolute exclusion was not observed, but there was a 3-day delay in CHIKV dissemination in the mosquitoes [108]. In *Ae aegypti*, ZIKV transmission could be blocked by prior infection with an insect-specific flavivirus [141].

In mosquitoes, it is unlikely that all cells will be infected with a first virus, leading to uninfected cells being available to be infected by a second virus. However, the situation in culture is very different, since superinfection exclusion requires a cell to be infected with a first virus before it can exclude a second one. Because of the high multiplicity of infection in vitro, most cells may be infected with a first virus, leading to effective exclusion of a second virus. The situation in vivo may be different if the mechanism of superinfection exclusion function without all cells needing to be infected by the first virus. In mosquitoes, as in plants, there is evidence that the RNAi defense system may be transmitted from infected cells to uninfected cells, with the potential to protect the whole organism [142]. Mosquitoes genetically modifying to express RNAi-activating sequences from pathogenic or insect-only viruses offer a possibility, enhanced by the finding that infection of insects with RNA viruses results in reverse transcription to DNA in a system that amplifies the RNAi response and provides both specificity and memory [143,144].

Understanding and characterizing the mechanisms behind superinfection exclusion is key in utilizing this phenomenon for controlling arboviruses. The use of superinfection exclusion as an arbovirus control strategy could, in theory, be viable but it presents several challenges. The mechanism by which one virus blocks another in a specific vector is quite complex. Superinfection exclusion seems very specific/restricted to mostly some viruses within the same family. There are several regulatory hurdles to be considered before implementing such strategy. Mosquitoes infected with low-pathogenicity arboviruses can still infect vertebrate hosts and, in some cases, they might cause mild disease. Insect-specific viruses could prove valuable here, but their transmission rates and their effect on mosquito fitness will need to be investigated. More detailed studies into the mechanisms of refraction between related viruses are needed.

Once these challenges are overcome, it could be plausible to infect local populations of mosquitoes with non-pathogenic viruses or with insect-specific viruses to reduce the prevalence of a pathogenic virus. The strategies for carrying this out are already being used for *Wolbachia* population replacement programs. This method would maintain local mosquito populations and the environment intact, without the need of pesticides or genetic manipulation. In addition, understanding the mechanisms of superinfection exclusion could allow us to develop new genetically engineered mosquitoes with lower fitness cost. This understudied research area is challenging but will provide a novel alternative to reduce the impact of arboviruses.

## Figures and Tables

**Table 1:** List of published papers reporting superinfection exclusion and co-infection phenomena in *Aedes* mosquito cells and *Aedes* mosquitoes [46,99,100,101,102,103,104,106,107,108,109,110]. Viruses: SINV, Sindbis, CHIV; chikungunya, DENV, dengue, SFV; RVV; Ross River, PCV, MVEV, WNV, West Nile, NHUV, SLEV, St. Louis encephalitis, JEV, Japanese encephalitis, VEEV, Venezuelan equine encephalitis, EEV, Eastern equine encephalitis, YFV, yellow fever, ZIKV, Zika.

Primary Infecting Virus (Family)	Observed Superinfection Exclusion	No Superinfection Exclusion	Cell Line	Mosquito Species	References
SINV (Alphaviridae)	SINV (Alphaviridae)	EEEV (Alphaviridae)	*Aedes albopictus*		[100]
SINV (Alphaviridae)	SINV (Alphaviridae) SFV (Alphaviridae) Una virus (Alphaviridae) CHIKV (Alphaviridae)	Snowshoe hare virus (Peribunyaviridae)	*Ae. albopictus*		[101]
La Crosse virus (Peribunyaviridae)	Snowshoe hare virus (Peribunyaviridae)			*Aedes triseriatus*	[106]
Snowshoe hare virus (Peribunyaviridae)	La Crosse virus (Peribunyaviridae)			*Ae. triseriatus*	
SINV (Alphaviridae)	Aura virus (Alphaviridae) SFV (Alphaviridae) RRV (Alphaviridae)	YFV (Flaviviridae)	C6/36, C7-10 and U4.4 (*Ae. albopictus*)		[102]
DENV-1 (Flaviviridae)		CHIKV (Alphaviridae)		*Ae. albopictus*	[103]
PCV (Flaviviridae)	MVEV (Flaviviridae) WNV (Flaviviridae)		C6/36 (*Ae. albopictus*)		[46]
NHUV (Flaviviridae)	WNV (Flaviviridae) JEV (Flaviviridae) SLEV (Flaviviridae)		C6/36 (*Ae. albopictus*)		[107]
EILV (Alphaviridae)	SINV (Alphaviridae) VEEV (Alphaviridae) EEEV (Alphaviridae)		17/10 (*Ae. albopictus*)		[108]
		CHIKV (Alphaviridae)		*Aedes aegypti*	
NHUV (Flaviviridae)	WNV (Flaviviridae)		C6/36 (*Ae. albopictus*)		[109]
DENV-2 (Flaviviridae)	DENV-2 (Flaviviridae) YFV (Flaviviridae)		C6/36 (*Ae. albopictus*)		[99]
YFV (Flaviviridae)	DENV-2 (Flaviviridae) YFV (Flaviviridae)				
ZIKV (Flaviviridae)		CHIKV (Alphaviridae)	C6/36 (*Ae. albopictus*)	*Ae. aegypti*	[104]
NHUV (Flaviviridae)	ZIKV (Flaviviridae) DENV-2 (Flaviviridae)	CHIKV (Alphaviridae)	C6/36 (*Ae. albopictus*)		[110]
